# Pre-existing immunity to heartland virus protects rhesus macaques from challenge with severe fever with thrombocytopenia syndrome virus

**DOI:** 10.1080/22221751.2026.2731506

**Published:** 2026-09-23

**Authors:** Thomas Bisom, Trenton Bushmaker, Natalie McCarthy, Jamie Lovaglio, Chad S. Clancy, Carl Shaia, Heinz Feldmann, David W. Hawman

**Affiliations:** a Laboratory of Virology, Division of Intramural Research, National Institute of Allergy and Infectious Diseases, National Institutes of Health, Rocky Mountain Laboratories, Hamilton, MT, USA; b Rocky Mountain Veterinary Branch, Division of Intramural Research, National Institute of Allergy and Infectious Diseases, National Institutes of Health, Rocky Mountain Laboratories, Hamilton, MT, USA; c Laboratory of Viral Zoonoses, Division of Intramural Research, National Institute of Allergy and Infectious Diseases, National Institutes of Health, Integrated Research Facility Frederick, Frederick, MD, USA

**Keywords:** SFTSV, HRTV, severe fever with thrombocytopenia syndrome, heartland virus, animal model

## Abstract

Heartland virus (HRTV) and Severe fever with thrombocytopenia syndrome virus (SFTSV) are closely related phenuiviruses that cause similar diseases in humans. However, animal disease models for both viruses are limited. Here, we first investigated whether human disease from SFTSV and HRTV could be recapitulated in rhesus macaques. In contrast to previous studies, we challenged rhesus macaques with a Japanese isolate of SFTSV. Although Japan reports higher human case fatality rates than many other regions, only mild disease was observed in infected rhesus macaques. Moreover, since animal models for HRTV are also limited, we challenged another group of rhesus macaques with HRTV. Although the animals seroconverted, clinical disease was not observed, suggesting rhesus macaques are unsuitable disease models for HRTV. Lastly, we subsequently tested whether immune responses raised against HRTV could cross-protect against SFTSV challenge and found that prior infection with HRTV limited SFTSV replication and signs of disease. Together, our data establishes a rhesus macaque model of SFTSV infection using a Japanese isolate of SFTSV and demonstrates that pre-existing immunity to HRTV can protect against infection with SFTSV.

## Introduction

Heartland virus (HRTV) and Severe fever with thrombocytopenia syndrome virus (SFTSV) are closely related tick-borne bandaviruses. Both are taxonomically classified under genus *Bandavirus*, in the family *Phenuiviridae* within the order *Hareavirales* in the class *Bunyaviricetes* [1]. Recently, SFTSV has also been reclassified as Dabie Bandavirus and is also known as Huaiyang Shan virus [2,3]. SFTSV is carried by the Asian Longhorned tick (*Haemaphysalis longicornis*) [4,5], and the tick-vector for HRTV is the Lone Star tick (*Amblyomma americanum*) [6,7]. Although cases of SFTS are currently limited to mostly Asian countries [8], the tick-vector for SFTSV is currently found in the eastern and midwestern United States, creating a risk for establishment of SFTSV in this country if introduced [9]. Furthermore, the Lone Star tick is also located in the United States, and the geographic range of the tick and HRTV cases continue to expand, with a recent fatal HRTV case reported in Maryland [10].

In addition to the high genetic similarity between HRTV and SFTSV, both viruses also cause similar disease with hallmark features such as thrombocytopenia and fever [11,12]. Symptoms from both viruses can progress to multi-organ dysfunction, sometimes leading to multi-organ failure and mortality [13–17]. There have been about 60 human cases of HRTV infection in the United States, with a case fatality rate of up to 10% [18]. Case fatality rates from SFTSV range from about 5 to 30% [8,19–21]. SFTSV case mortalities have been shown to vary based on region, with Japan reporting a higher case fatality rate than China [3]. While this could be due to differences in public health systems, studies have reported that SFTSV isolates from regions reporting higher case fatality rates are more pathogenic in the limited animal challenge models, suggesting there may be strain-dependent effects on SFTSV pathogenicity. For example, when aged ferrets were inoculated with different isolates of SFTSV from South Korea, survival varied based on the SFTSV genotype [22]. Human case fatality rates in China have also been shown to vary based on the clade of SFTSV [23]. Collectively, these results suggest severity of disease in both humans and animal models could vary based on the strain of SFTSV.

Animal disease models for HRTV and SFTSV are currently limited. Only mice lacking both type I and II interferon (IFN) receptors have been shown to be a fatal model system for HRTV [24,25]. Wild-type mice, chickens, hamsters, goats, rabbits and raccoons did not develop viremia or clinical signs of disease upon HRTV infection [25]. More animal models have been assessed for SFTSV. Newborn mice and mice with impaired type I IFN interferon responses (IFNAR^−/−^ and MAR15A3-treated mice) have been shown to be lethal disease models for SFTSV infection [26–28]. Furthermore, limited immunocompetent animal disease models have also been reported, with aged ferrets shown to recapitulate lethal disease upon challenge with SFTSV [29]. Domestic cats and dogs can also develop severe disease during infection with SFTSV [30,31], but the use of companion animals in research is ethically limited. Non-human primates (NHPs), such as rhesus macaques and cynomolgus macaques, have been previously assessed as disease models for SFTSV, and although some human disease parameters have been recapitulated in rhesus macaques, lethality has not been observed in NHPs [32,33]. However, given evidence of strain-dependent effects on pathogenicity in other animal models [22], the less severe disease observed may have been due to the strains of SFTSV that were used in those studies. Furthermore, HRTV has also been tested in cynomolgus macaques, but no disease symptoms were observed [32].

Here, due to higher case fatality rates being reported in Japan, we hypothesized that challenge of rhesus macaques with a Japanese isolate of SFTSV, SFTSV SPL010A [34], would result in a more severe disease course, providing a more suitable model for study of countermeasures against SFTSV. For comparison, we challenged another cohort of rhesus macaques with a Chinese isolate of SFTSV, SFTSV YL-1. We challenged a third cohort of rhesus macaques with HRTV as to our knowledge, rhesus macaques have not been evaluated as a potential disease model for HRTV. Lastly, based on the antigenic and genetic similarities between SFTSV and HRTV, we evaluated whether prior infection with HRTV could confer protection against SFTSV challenge.

## Results

### Challenge of rhesus macaques with SFTSV SPL010A results in mild disease

To determine if a Japanese isolate of SFTSV, SFTSV SPL010A, would result in severe disease upon inoculation of rhesus macaques, we challenged one female and two male rhesus macaques with SFTSV SPL010A (Supplemental Table 1). Each NHP was infected with 1 × 10^6^ TCID_50_ of SFTSV SPL010A, and the target dose was confirmed by back-titrations. All NHPs were infected by combined subcutaneous and intravenous routes, with 1 mL inoculum per route, similar to our established cynomolgus macaque infection model for the distantly related Crimean-Congo haemorrhagic fever virus [35]. Exams with blood draws were performed on 0, 1, 3, 5, 7, 10, 14, 21, and 28 days post-infection (DPI), and the NHPs were also comprehensively monitored daily for clinical signs of disease. None of the NHPs met euthanasia criteria by 28 DPI.

Despite no animals achieving euthanasia criteria, we measured elevated temperature and viremia in infected animals. In all three SPL010A-infected macaques, temperatures were elevated until 7 DPI, at which point they began returning to baseline levels (Figure 1(a)). Viremia peaked in two of the three macaques on 3 DPI and on 5 DPI in the remaining macaque (Figure 1(b)). Peak viremia of >10^5^ TCID_50_ was seen in all animals. Moreover, one of the NHPs developed a pale appearance, and another had reduced faeces and eating, suggestive of mild clinical disease (Figure 1(c)). Blood chemistry was also consistent with mild clinical disease. Thrombocytopenia, indicated by decreased platelet (PLT) levels, and liver pathology, indicated by elevated aspartate transaminase (AST) and alanine aminotransferase (ALT) levels, often occur in severe human cases of SFTSV infection [16,36–38]. We measured mild increases in AST and ALT and drop in platelets in one SFTSV-infected animal that peaked at 7 DPI (Figure 1(d–f)). Interestingly, the NHP exhibiting these blood chemistries was older in age (∼16 years of age) than the other two animals (∼4 and ∼6 years of age). SFTSV is known to be more severe in older humans [39,40] and is also lethal in aged ferrets [29].

**Figure 1.**
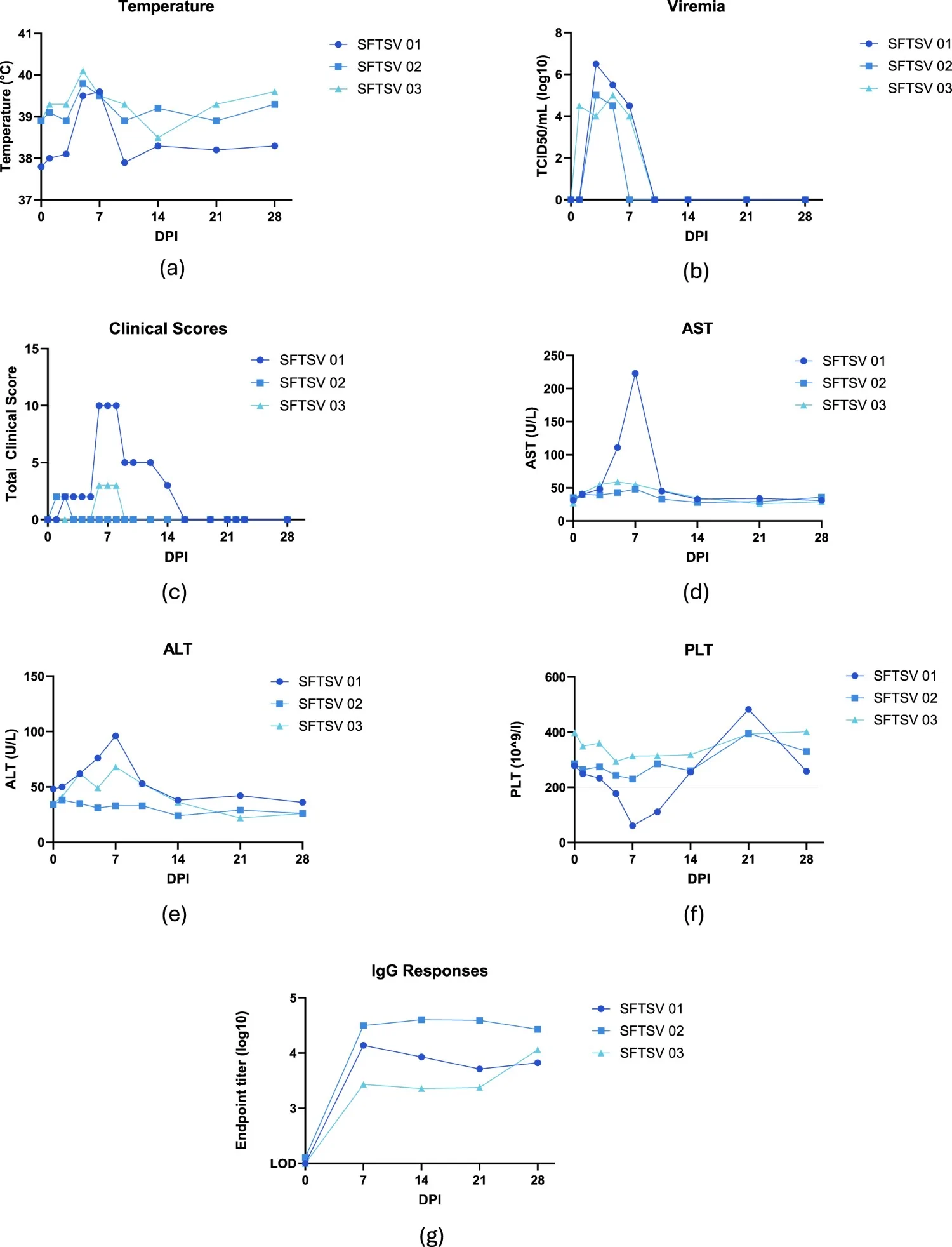
Infection of rhesus macaques with SFTSV SPL010A results in mild clinical disease. Three rhesus macaques were each infected with ∼1 × 10^6^ TCID_50_ SFTSV SPL010A by combined IV/subcutaneous routes. Temperature (a), viremia (b), clinical scores (c), AST (d), ALT (e), and platelet count(PLT) (f) were measured as readouts for disease parameters. Antibody responses to SFTSV whole-virus antigen to confirm infection were performed using ELISA (g). Individual values are plotted to show variability in the parameters between the NHPs of each group.

Antibody responses against SFTSV were measured by ELISA and showed that all animals had SFTSV-specific IgG by 7 DPI, supportive of the animals being infected with SFTSV (Figure 1(g)). Interestingly, we largely did not measure an increase in SFTSV-specific IgG from 7 to 28 DPI, suggesting peak SFTSV-specific humoral responses were rapidly achieved after infection. Appearance of SFTSV-specific IgG on 7 DPI correlated with return to baseline of most parameters and rapid decreases in viremia.

To determine if a Chinese isolate of SFTSV, strain YL-1, performed similarly in our institution as previous reports and to establish a second model with a distinct strain of SFTSV, we also infected two additional rhesus macaques by the combined subcutaneous and intravenous routes with SFTSV YL-1 (Supplemental Table 1). Similar to the infection with SFTSV SPL010A, each NHP was inoculated with 1 × 10^6^ TCID50 SFTSV YL-1, which was confirmed with back-titrations. Although our group sizes were small, disease was largely similar in animals infected with SFTSV strain YL-1 versus those challenged with strain SPL010A (Supplemental Figure 1). Interestingly, the older rhesus macaque (∼17 years of age) infected with SFTSV YL-1 also displayed elevated AST and ALT levels (Supplemental Figure 1(d,e)), similar to what we observed in the older rhesus macaque infected with SFTSV SPL010A (∼16 years of age, Figure 1(d,e)). However, although platelets decreased in the older YL-1-infected macaque, the decrease was not as strong as that observed in the older macaque infected with SFTSV SPL010A (Supplemental Figure 1(f)). Overall, these results suggest infection of rhesus macaques with SFTSV SPL010A and SFTSV YL-1 resulted in similar mild disease.

### HRTV challenge of rhesus macaques does not result in disease

We previously observed differences in disease severity between cynomolgus and rhesus macaques challenged with CCHFV [35], and although cynomolgus macaques infected with HRTV showed no disease [32], we hypothesized that rhesus macaques could show signs of disease upon infection with HRTV. We challenged one female and two male rhesus macaques with ∼1 × 10^5^ TCID_50_ HRTV by combined subcutaneous and intravenous routes (Supplemental Table 1). While we intended to inoculate the animals with a similar dose as our SFTSV studies (1 × 10^6^ TCID_50_), back-titration of the inoculum by TCID_50_ assay indicated a lower than intended titre of ∼1 × 10^5^ TCID_50_. Similar to the NHPs infected with SFTSV, exams with blood draws were performed on 0, 1, 3, 5, 7, 10, 14, 21, and 28 DPI, and daily monitoring for clinical signs of disease was performed.

No animals reached euthanasia criteria, and unlike the rhesus macaques challenged with SFTSV, notable changes in temperature and viremia were not detected (Figure 2(a,b)). Also, unlike the infections with SFTSV, no clinical signs of disease were observed (Figure 2(c)). Furthermore,changes in AST, ALT, and PLT levels were minimal (Figure 2(d–f)). However, all NHP infected with HRTV developed HRTV-specific IgG, suggesting that although we measured little-to-no viremia, the NHPs were still productively infected with HRTV (Figure 2(g)). Collectively, these results suggest infection of rhesus macaques with HRTV results in limited viral replication and no overt disease.

**Figure 2.**
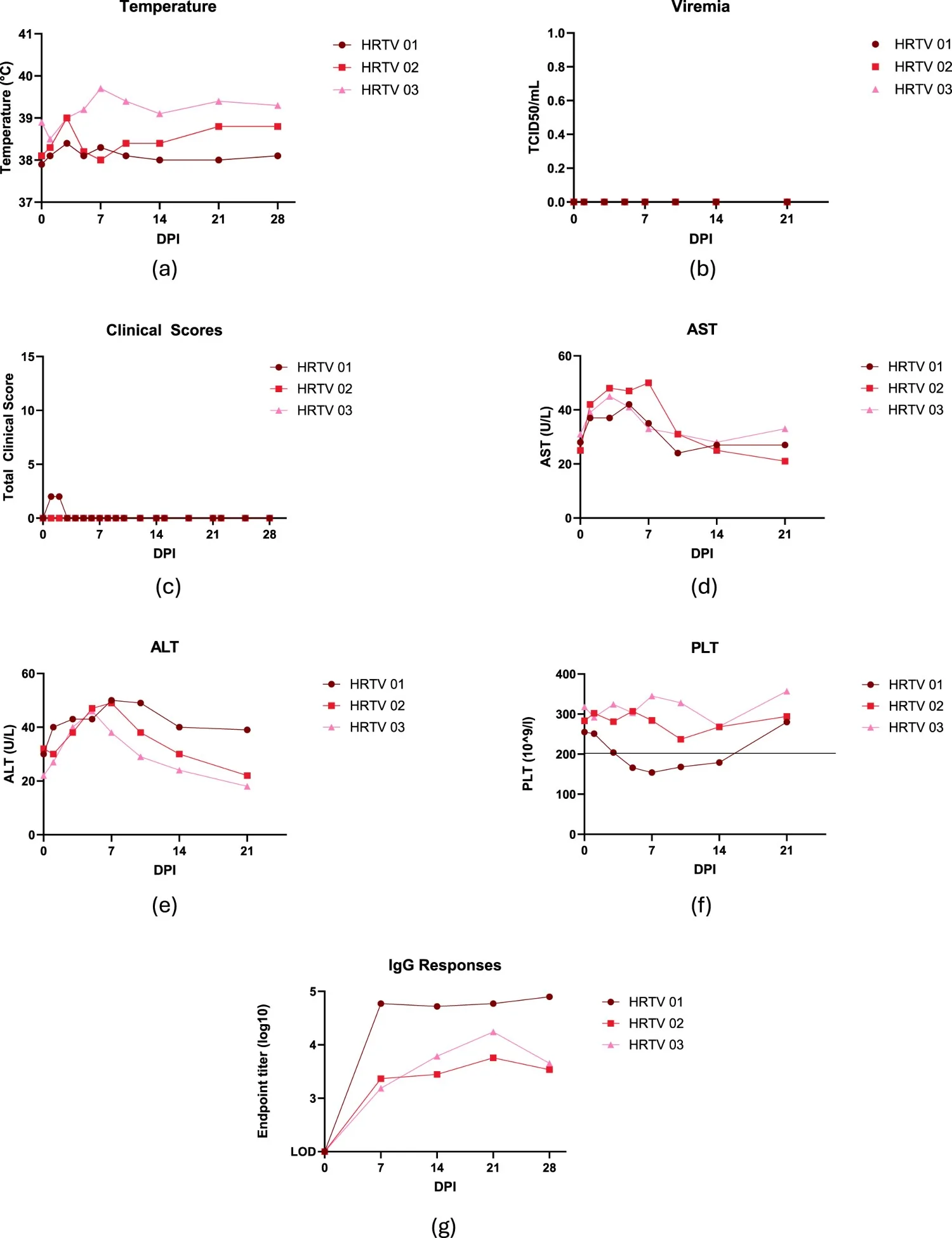
Infection of rhesus macaques with HRTV results in no disease. Three rhesus macaques were infected with ∼1 × 10^5^ TCID50 HRTV MO-4 by combined IV/subcutaneous routes. Temperature (a), viremia (b), clinical scores (c), AST (d), ALT (e), and PLT (f) were measured. Antibody responses to HRTV whole-virus antigen to confirm infection were performed using ELISA (g). Individual values are plotted to show variability in the parameters between the NHPs of each group.

### HRTV infection cross-protects against SFTSV challenge in rhesus macaques

Due to the genetic similarity between HRTV and SFTSV (L protein = ∼73%, GPC = ∼63%, small non-structural protein = ∼63%, nucleoprotein = ∼63% identical amino acids [41,42]), we then wondered if adaptive immune responses produced from the HRTV infection could cross-protect against SFTSV challenge. Sera of animals infected with HRTV cross-reacted with SFTSV whole-virus antigen (Figure 3(a)), and sera of NHPs infected with SFTSV cross-reacted with HRTV whole-virus antigen (Figure 3(b)), suggestive of cross-reactive humoral responses. We therefore challenged the three HRTV-infected NHPs with SFTSV SPL010A to evaluate protection from disease. Furthermore, as a comparison, we also challenged the three SFTSV-SPL010A-infected NHPs again with SFTSV SPL010A (Figure 3(c)). All six NHPs were each inoculated with 1 × 10^6^ TCID_50_ SFTSV SPL010A by combined subcutaneous and intravenous routes, and this dose was confirmed in back-titrations. We used data from the first challenge of the three NHPs with SFTSV SPL010A as historical controls for disease parameters in naïve animals. In the re-challenge, exams with blood draws were again performed on 0, 1, 3, 5, 7, 10, 14, 21, and 28 DPI. Similar to infection of naïve animals, none of the re-challenged NHPs met euthanasia criteria throughout this period. At study endpoint on re-challenge 28 DPI, the animals were euthanized, and no pathology likely associated with SFTSV was observed in the tissues, as expected due to the animals being euthanized past the timeframe of peak disease (Supplemental File).

**Figure 3.**
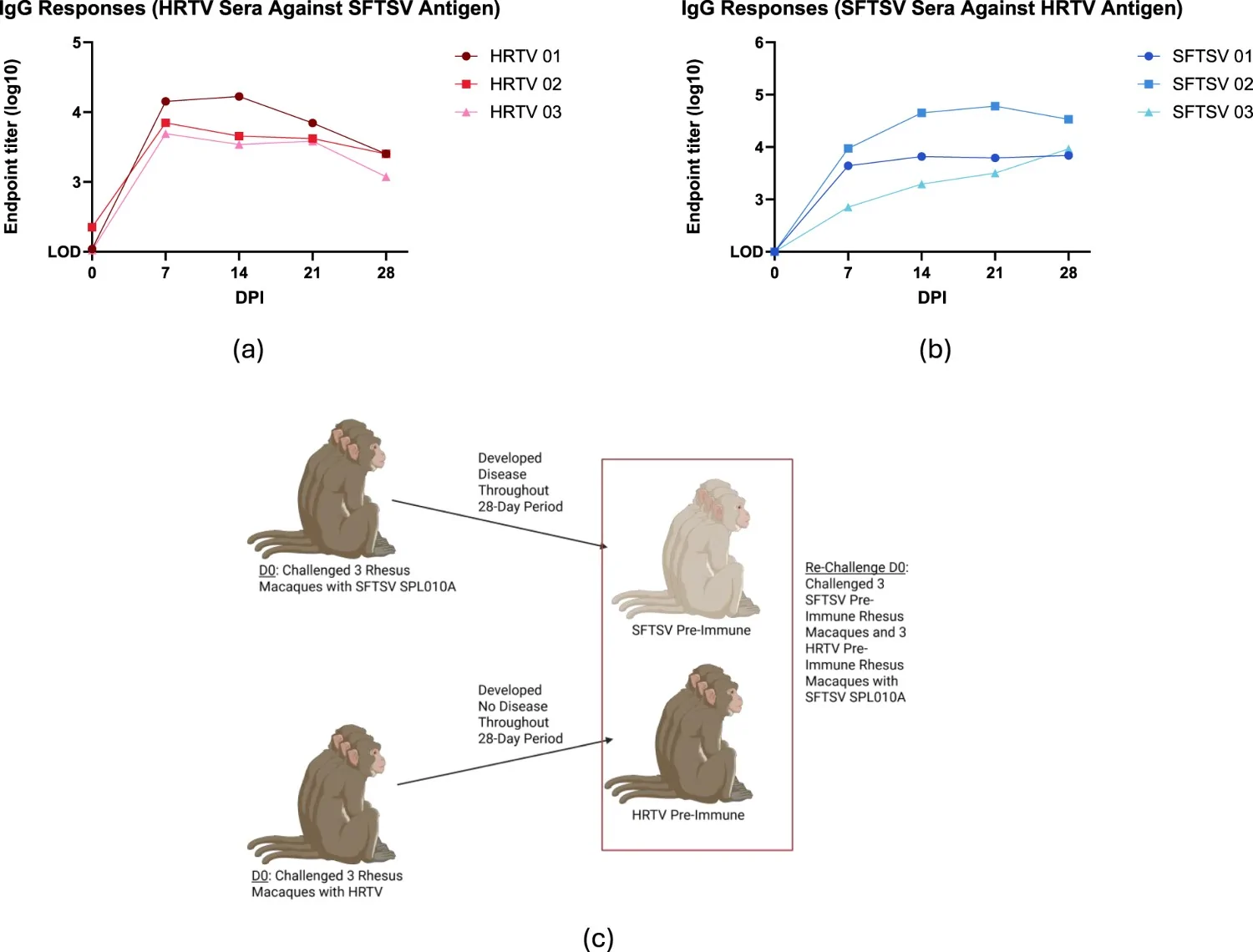
Rhesus macaque antibodies against HRTV cross-react with SFTSV whole-virus antigen and antibodies against SFTSV cross-react with HRTV whole-virus antigen. Prior to re-challenge, sera from each group demonstrated cross-reactivity, as indicated by ELISA. Sera from each HRTV-infected rhesus macaque cross-reacted with SFTSV whole-virus antigen (a), and sera from each SFTSV-infected rhesus macaque cross-reacted with HRTV whole-virus antigen (b). Due to evidence of cross-reactivity, the three rhesus macaques infected with SFTSV SPL010A (SFTSV Pre-Immune) and the three rhesus macaques infected with HRTV MO-4 (HRTV Pre-Immune) were then each re-challenged with ∼10^6^ TCID_50_ SFTSV SPL010A by combined IV/subcutaneous routes (c).

In contrast to naïve animals challenged with SFTSV SPL010A, SFTSV- or HRTV-pre-immune animals were protected against SFTSV challenge exhibiting no changes to temperature, no detectable viremia, and no observable clinical signs of disease (Figure 4(a–c)). Serum neutralization activity against SFTSV SPL010A was also assessed using sera from animals both before and after the re-infection. Prior to re-challenge, neutralization was only observed 28 DPI in animals infected with SFTSV SPL010A, but the VN titre was relatively low (Figure 4(d)). However, neutralization activity increased following re-challenge with SFTSV in animals previously infected with SFTSV SPL010A and also in animals previously infected with HRTV (Figure 4(d)).

**Figure 4.**
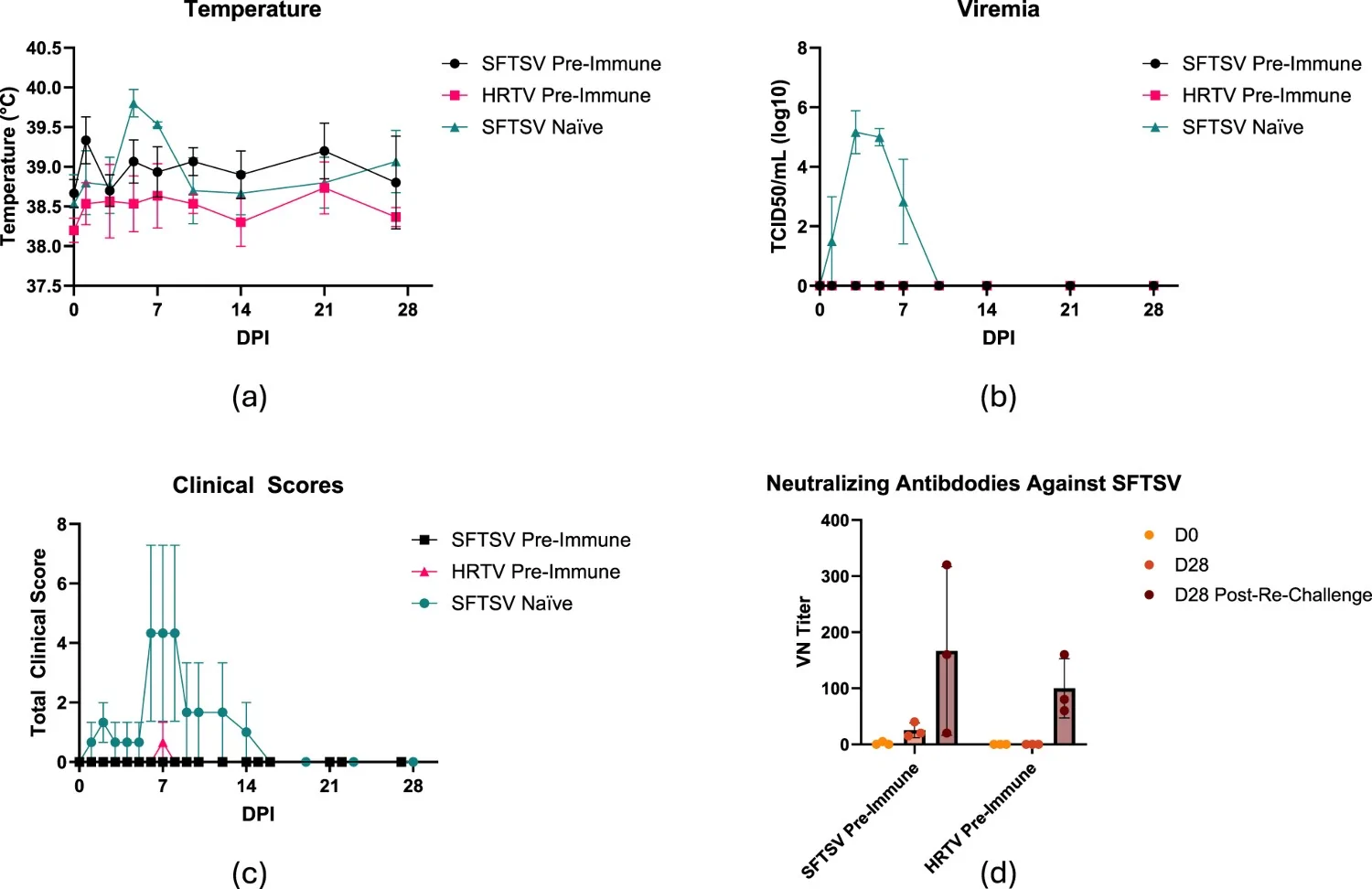
Prior HRTV infection in rhesus macaques prevents disease upon SFTSV infection. Rhesus macaques previously infected with SFTSV SPL010A or HRTV were re-challenged with SFTSV SPL010A . To compare differences in disease parameters following re-challenge, data from first infection of the three rhesus macaques were used as historical controls (SFTSV Naïve). Temperature (a), viremia (b), and clinical scores (c) were lower in the re-infected groups compared to historical controls. Neutralizing antibodies against SFTSV SPL010A, as measured by microneutralization assays, were also present in the re-infected animals (d). Variability in (a-c) is reported as standard error of the mean, and in (d) is reported as standard deviation.

Increased MCP-1, IFN-γ, IL-6, and TNF-α have all been associated with fatal human cases of SFTSV infections [43]. We therefore measured protein levels of various cytokines and chemokines in the sera of SFTSV-infected naïve (historical controls) and pre-immune NHPs. Following SFTSV infection of naïve animals, increases in IFN-γ, BLC (CXCL-13), IL-6, and TNF-α were observed. Consistent with protection from challenge, these cytokines were not elevated in SFTSV and HRTV pre-immune animals challenged with SFTSV (Figure 5(a–d)). A modest increase in MCP-1 was measured in SFTSV-infected naïve animals, but the fold-increases were relatively low (∼4–6 fold; Supplemental Figure 2(a)). Furthermore, BLC is a chemoattractant for B cells, which have been shown to be targeted by SFTSV in lethal human cases [44]. Moreover, macrophages are important sources of inflammatory cytokines, such as IL-6 and TNF-α, and changes in population sizes of these cells can be indirectly measured through changes in their circulating precursor cells, monocytes [45]. In conjunction with increased inflammatory cytokines, i.e. IL-6 and TNF-α, we also observed an increase in percentage of circulating monocytes in white blood cell counts in historical controls, most notably at 7 DPI, but this was not observed in SFTSV-challenged pre-immune animals (Figure 5(e)). Other chemokines, such as IL-8, IP-10, MIP-1b, and SDF-1a were also measured, but no considerable fold-changes from D0 were observed, aside from IL-8 where NHPs re-challenged with SFTSV interestingly showed higher levels compared to naïve animals during the first SFTSV infection (Supplemental Figure 2(b–e)). ITAC was also measured but was not detected.

**Figure 5.**
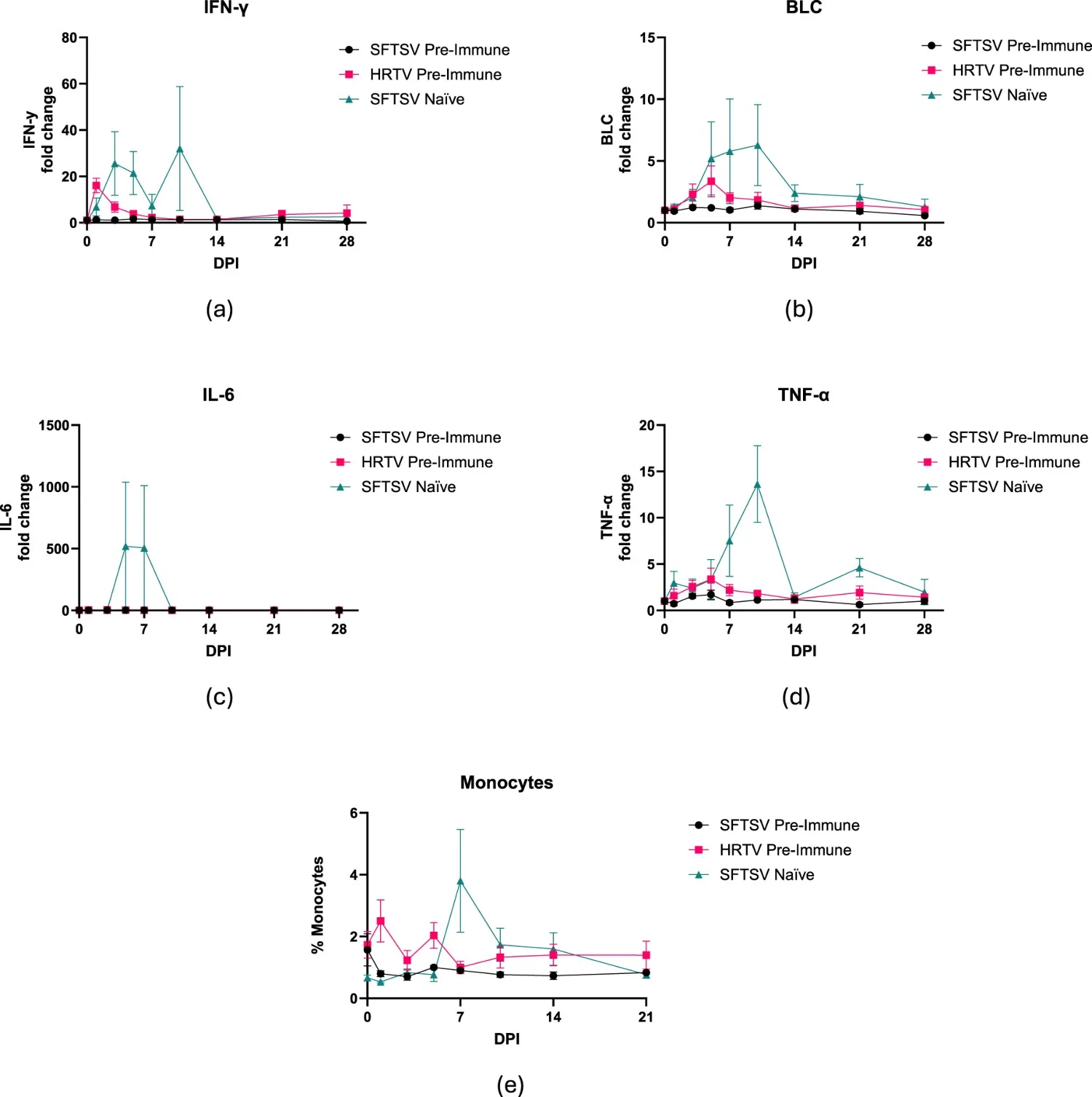
Inflammatory cytokines associated with severe disease in human SFTS cases were lower in both SFTSV pre-immune and HRTV pre-immune groups compared to historical controls. Fold-inductions from D0 of IFN-γ (a), BLC/CXCL-13 (b), IL-6 (c), and TNF-α (d) were considerably lower in re-infected macaques compared to those measured in historical controls. % Monocytes in white blood cell counts were also lower in SFTSV- and HRTV-pre-immune groups compared to historical controls, most notably at 7 DPI (e). Variability in (a-e) is reported as standard error of the mean.

## Discussion

Together, our data indicate that SFTSV infection of rhesus macaques with SFTSV strain SPL010A recapitulates mild clinical disease. While a model demonstrating consistent severe or lethal disease would be ideal for evaluation of countermeasures against SFTSV, we still measured consistent viremia, fever, and inflammatory cytokine responses in infected animals that would provide readouts for countermeasure efficacy. In contrast to the mild disease in infected NHPs, aged ferrets consistently develop severe disease [29] and may be more suitable for testing vaccines and other countermeasures against severe SFTS. Still, the genetic similarities between humans and NHPs may warrant the need to test countermeasures in NHPs. For example, we have previously reported marked differences in responses to a CCHFV vaccine in mice and NHPs [46], suggesting variation in responses to bunyavirus vaccines across animal species. Further, our study examining SFTSV infection in NHPs with pre-existing immunity elicited by HRTV or SFTSV infection demonstrates proof-of-concept that the rhesus macaque model provides sufficient parameters to measure the impact of countermeasures, such as vaccines. Alternative NHP species such as marmosets and/or African green monkeys could also have potential for being suitable NHP disease models for SFTSV and/or HRTV infections, since they have been shown to be lethal models for Rift Valley fever virus, which is in the same family as SFTSV and HRTV (*Phenuiviridae*) [47,48].

Our data supports previous reports of the antigenic similarity between SFTSV and HRTV. Other groups have demonstrated that vaccinating against SFTSV can cross-protect against HRTV challenge in mice. This has been shown with an mRNA vaccine encoding SFTSV Gn/Gc [49] and also with a recombinant Vesicular stomatitis virus vaccine vector expressing SFTSV Gn/Gc [50]. Here, we show the converse: immune responses raised against HRTV can cross-protect against SFTSV and confirm this antigenic cross-reactivity extends to NHPs. We also found that sera from NHPs infected only with SFTSV cross-reacted with HRTV whole-virus antigen (Figure 3(b)). Furthermore, in comparison to other studies involving SFTSV/HRTV cross-protection, our study examined immunity generated from prior infection instead of vaccine-elicited immunity, which likely raised more broad immune responses against a variety of antigens from HRTV. Together, our data and data from previous vaccine studies in mice support the notion that SFTSV and HRTV are sufficiently related such that vaccines against either will confer protection against the heterologous virus, likely simplifying clinical development.

Although our study was not powered to appropriately investigate whether age of the rhesus macaques influences disease, we did see a trend towards more severe disease in the older macaques. The oldest NHPs infected with SFTSV SPL010A (∼16 years of age) and YL-1 (∼17 years of age) both exhibited higher viremia, increased clinical scores, elevated AST and ALT levels, and decreased platelets compared to the other NHPs in each group, all indicative of more pronounced disease parameters. Given that aged ferrets have been shown to model severe SFTS [29] and that increased age is a correlate of poor outcome in SFTSV infected humans [39,40], these results suggest age could influence the severity of SFTS in rhesus macaques. Future studies with larger cohorts of aged and younger animals are warranted. However, since each of these NHPs were also the only females in each group, we cannot exclude the possibility of biological sex playing a role in the more pronounced disease parameters.

Our study has some limitations. First, NHPs infected with SFTSV only developed mild disease, which limits investigations of the pathogenesis of more severe outcomes of SFTSV infection. However, the study identified consistent clinical disease parameters, allowing the rhesus macaque to serve as a valuable non-lethal infection model whereby, similar to the CCHFV NHP model, samples can be taken at the height of clinical disease instead of following the animals for survival. Further, subclinical infections with SFTSV may be common [51–53], and NHPs may accurately model this underappreciated outcome of human SFTSV infections. Second, while we cannot exclude the possibility that a higher inoculation dose of HRTV may result in greater disease, challenge of rhesus macaques with 10^5^ TCID_50_ HRTV resulted in no overt signs of disease and little-to-no viremia, similar to previous studies in cynomolgus macaques. This makes the model unsuitable for evaluating pathogenicity and efficacy of countermeasures against HRTV. Lastly, although we measured some differences in disease severity between animals infected with different strains of SFTSV and between the younger and older animals in our study, our studywas not sufficiently powered to identify smaller differences in disease severity. Thus, further studies with larger cohorts will be needed to conclusively determine whether strain of SFTSV or age of NHPs influences overall disease severity in this model.

Cumulatively, we show that SFTSV challenge of rhesus macaques models mild disease reported in humans but provides parameters such as consistent viremia, fever, and thrombocytopenia with which to evaluate countermeasures against SFTSV. Moreover, we found rhesus macaques are resistant to HRTV infection, and along with previous studies evaluating HRTV infection in cynomolgus macaques, this suggests further work will be needed to develop a non-mouse model of disease for HRTV. Although no disease was observed in rhesus macaques infected with HRTV, we found immune responses elicited by HRTV were able to cross-protect against SFTSV infection, confirming the antigenic relatedness between these viruses in a NHP infection model. Even though our study highlights the current limitations in NHP disease models for SFTSV and HRTV, it supports the possibility that vaccination with HRTV or SFTSV antigens will likely cross-protect against heterologous virus infections, providing a simpler path for vaccine development against both pathogens.

## Methods

### Animals, biosafety, and ethics

All infectious animal work with SFTSV and HRTV was performed in high or maximum containment at Rocky Mountain Laboratories (RML), Division of Intramural Research (DIR), NIAID, NIH as previously described [46]. Laboratory procedures and sample inactivations were performed in accordance with standard operating procedures approved by the RML Institutional Biosafety Committee. All animal work was performed in strict accordance with the recommendations described in the Guide for the Care and Use of Laboratory Animals of the Office of Animal Welfare, National Institutes of Health and the Animal Welfare Act of the US Department of Agriculture, in an AAALAC-accredited facility according to Rocky Mountain Laboratories Animal Care and Use Committee (ACUC) approved protocols. NHPs were housed in adjoining individual primate cages, which prevented social isolation. Environmental conditions of the cages, such as humidity, temperature, and light (12-h light/12-h dark cycles), were carefully controlled. Water was also steadily provided. Animals were monitored at least twice daily and fed twice a day by trained personnel. Upon infection, animals were comprehensively evaluated for disease signs using a score sheet approved by the ACUC. Environmental enrichment consisted primarily of visual and audio stimulation. All procedures on NHPs were performed by board-certified clinical veterinarians who also provided veterinary oversight of the study.

### Viral stocks

SFTSV SPL010A was kindly provided by the Laboratory of Arboviruses, National Institute of Infectious Diseases in Tokyo, Japan. The virus was grown on Vero E6 cells to generate a working stock, and both the original and working stock were sequenced to confirm there were no mutations during propagation. Sequencing also confirmed absence of mycoplasma and other contaminants. The stocks were titered on Vero E6 cells using an immunofluorescence TCID_50_ assay described below [54,55].

SFTSV strain YL-1 was kindly provided by the World Reference Center for Emerging Viruses and Arboviruses at the University of Texas Medical Branch and cleared of Mycoplasma as described previously [28]. No mutations or contaminants were found upon sequencing of final stocks. The final stocks were titered on Vero E6 cells using an immunofluorescence TCID_50_ assay described previously [28,54,55] (and below).

HRTV M0-4 was grown on Vero E6 cells and sequenced. No mutations or contaminants were found. Final stocks were titered on Vero E6 cells using a standard TCID_50_ assay.

### TCID_50_ infectious assays

Infectious SFTSV (SPL010A) was measured by TCID_50_ assays utilizing immunofluorescence (IFA) as described previously [28,54,55] and TCID50/mL values were calculated using the Reed-Muench method. Briefly, samples were diluted 10-fold, applied to Vero E6 cells, and fixed with formalin five days later. Immunofluorescence assays were then performed using primary human antibodies targeting SFTSV Gn (Creative Biolabs) and AlexaFluor goat anti-human secondary antibodies (Invitrogen) for detection of internalized SFTSV. Antibodies were incubated with cells in permeabilization solution consisting of PBS, 0.05% Saponin, and 0.1% BSA. Cells were then visualized and scored for infectious virus on a ZOE Fluorescent Cell Imager (BioRad).

Infectious HRTV was measured using TCID_50_ assays. Vero E6 cells were seeded onto 96-well plates, and HRTV samples were diluted 10-fold down each plate. Five days later, wells were scored for cytopathic effects, and TCID_50_/mL was subsequently calculated using the Reed-Muench method.

### Neutralization assays

Neutralization assays were performed as described previously [28]. Sera samples were diluted 2-fold and incubated with 120 TCID_50_ SFTSV for 1 h. These samples were then applied to Vero E6 cells, incubated for 5 days, and infectious virus was quantified using the TCID_50_/IFA method described above.

### ELISA

For the whole-virus SFTSV and HRTV antigen ELISAs, antigens were prepared by infecting Vero E6 cells with either virus and collecting supernatant at 5 DPI. The virus was then pelleted by ultracentrifugation through a 20% sucrose cushion. The viral pellet was resuspended in 4 ml PBS + 2% Triton-X 100 and irradiated with 10 megarad (Mrad) of γ-radiation by a JL Shepherd Model 484 irradiator with a cobalt cell. SFTSV antigen was adsorbed at 1:500 and HRTV antigen at 1:2000 in PBS to Nunc Maxisorp plates overnight at 4°C. After blocking with 5% skim milk and washing the plates, NHP sera samples were diluted 4-fold, beginning with 1:100 dilutions, and then applied to the Nunc Maxisorp plates for 1 h. Plates were washed thoroughly, and then, a Goat Anti-Monkey IgG H&L (HRP) secondary antibody (Abcam) in blocking buffer was applied at a 1:2000 concentration for 1 h incubation. Plates were washed again, and then, they were developed with 2, 2′-azino-di(3-ethylbenzthiazoline-6-sulfonate) for 30 min, which was terminated with 5% sodium dodecyl sulphate (Sigma) in water. Absorbance at 405 nm was measured. To calculate endpoint titres, a cut-off value for determining when samples were not exhibiting binding to antigen was first calculated. The cut-off value was equivalent to the average value of the blank wells (i.e. wells with no sample) plus three standard deviations. Dilutions were then converted to logarithmic values (base 10), and nonlinear regression (sigmoidal, 4PL) was then performed with unknowns interpolated from the standard curve . The antilog of values corresponding to cut-off values were then calculated, and the inverse of those values were reported.

### Cytokine assays

ProcartaPlex™ NHP Chemokine Panel (Thermo Fisher; Cat. EPX100-40041-901) capture beads were combined with beads from simplex panels for IL-6 (Thermo Fisher; Cat.EPX01A-40213-901), TNF-α (Thermo Fisher; Cat. EPX01A-40223-901), and IFN-γ (Thermo Fisher; Cat. EPX01A-40228-901). Beads were added to a 96-well plate and washed 2x with wash buffer. Serum samples were diluted 1:1 using universal assay buffer and added to the plate alongside a four-fold serial dilution using the provided standards. After a two-hour incubation, the plate was washed twice using wash buffer. Bound antibody was detected using the provided secondary antibody, and after a thirty-minute incubation, the plate was washed using wash buffer twice. Streptavidin-PE was applied, and the plate was incubated for thirty minutes prior to washing with wash buffer twice. Samples were resuspended in wash buffer prior to analysis. Analysis was performed using the MAGPIX system (Luminex) and Luminex xPONENT for MAGPIX software, and the cytokine concentration of samples was calculated using the ProcartaPlex Analysis App on Thermo Fisher.

### Statistics

Statistics were performed using GraphPad Prism 11.

## Supplementary Material

Supplemental Material

Supplemental_Table1.docx

Supplemental File.xlsx

## Data sharing

Raw data, including exam data, are available at https://doi.org/10.6084/m9.figshare.32411856.
