# Supplementary material for: Pre-existing immunity to heartland virus protects rhesus macaques from challenge with severe fever with thrombocytopenia syndrome virus
Source: Emerg Microbes Infect. 2026 Sep 23;15(1):2731506. doi: 10.1080/22221751.2026.2731506 (PMC13618116; doi:10.1080/22221751.2026.2731506)
Supplement: Supplementary file 1 [file TEMI_A_2731506_SM4954.docx]

| NHP Study ID | **Challenge Virus** | **NHP Sex** | **NHP Age** | **NHP Starting Weight** |
| --- | --- | --- | --- | --- |
| **SFTSV 01** | SFTSV SPL010A | Female | ~16.5 Years | 9.63 kg |
| **SFTSV 02** | SFTSV SPL010A | Male | ~5.9 Years | 8.45 kg |
| **SFTSV 03** | SFTSV SPL010A | Male | ~4.2 Years | 6.11 kg |
| **SFTSV 04** | SFTSV YL-1 | Female | ~17.0 Years | 8.72 kg |
| **SFTSV 05** | SFTSV YL-1 | Male | ~ 3.8 Years | 6.92 kg |
| **HRTV 01** | HRTV MO-4 | Female | ~15.9 Years | 7.68 kg |
| **HRTV 02** | HRTV MO-4 | Male | ~15.2 Years | 15.40 kg |
| **HRTV 03** | HRTV MO-4 | Male | ~4.1 Years | 8.17 kg |
