# Supplementary material for: Pre-existing immunity to heartland virus protects rhesus macaques from challenge with severe fever with thrombocytopenia syndrome virus
Source: Emerg Microbes Infect. 2026 Sep 23;15(1):2731506. doi: 10.1080/22221751.2026.2731506 (PMC13618116; doi:10.1080/22221751.2026.2731506)
Supplement: Supplementary file 2 [file TEMI_A_2731506_SM1403.zip › Supplemental Figure 1.pdf]

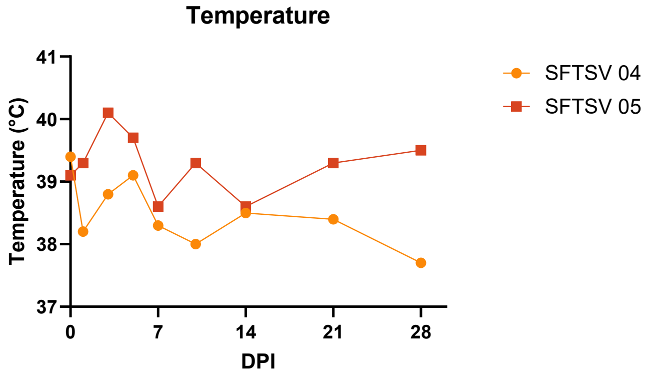
Temperature
Temperature (°C)
41
40
39
38
37
0
7
14
21
28
DPI
SFTSV 04
SFTSV 05

(a)

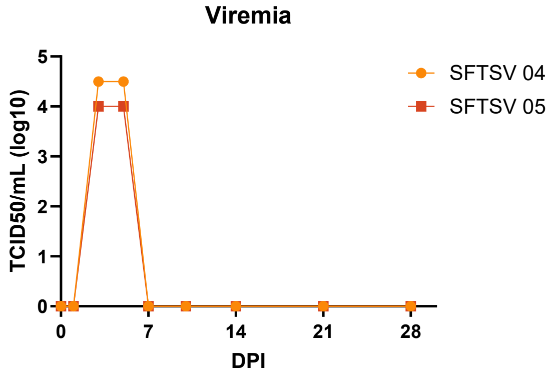
Viremia
TCID50/mL (log10)
5
4
3
2
1
0
0
7
14
21
28
DPI
SFTSV 04
SFTSV 05

(b)

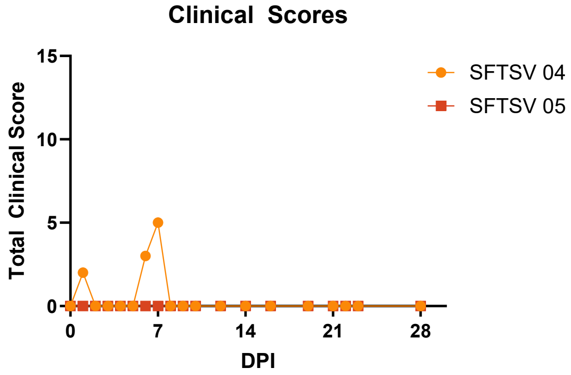
Clinical Scores
Total Clinical Score
15
10
5
0
0
7
14
21
28
DPI
SFTSV 04
SFTSV 05

(c)

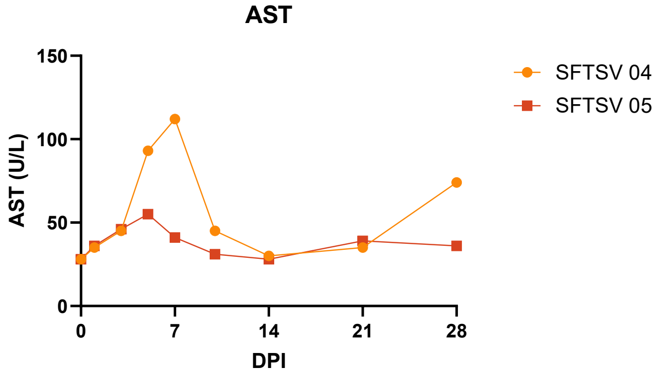
AST
AST (U/L)
150
100
50
0
0
7
14
21
28
DPI
SFTSV 04
SFTSV 05

(d)

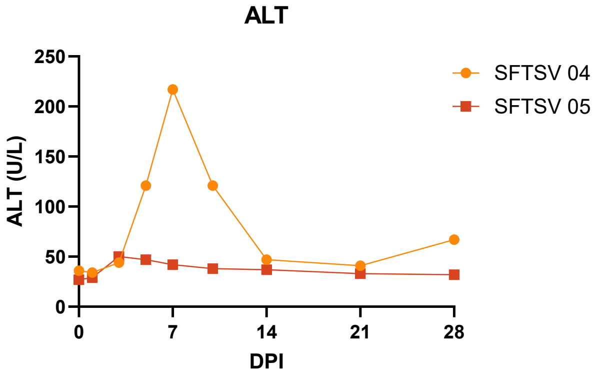
ALT
ALT (U/L)
250
200
150
100
50
0
0
7
14
21
28
DPI
SFTSV 04
SFTSV 05

(e)

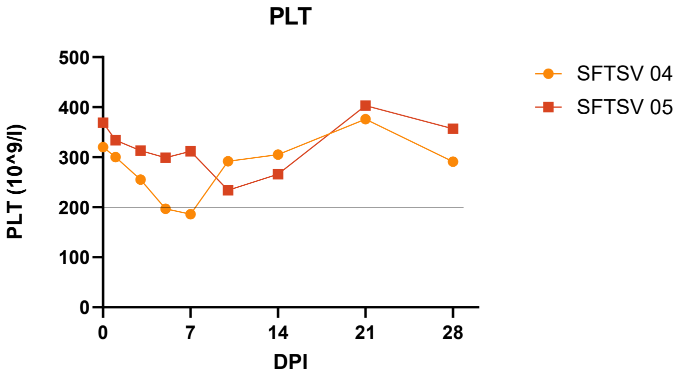
PLT
PLT (10^9/l)
500
400
300
200
100
0
0
7
14
21
28
DPI
SFTSV 04
SFTSV 05

(f)

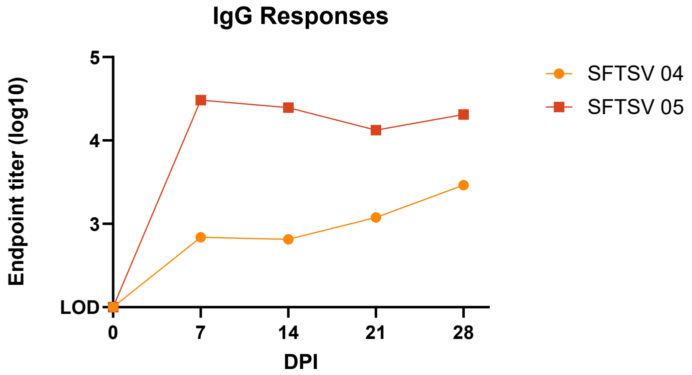
IgG Responses
Endpoint titer (log10)
5
4
3
LOD
0
7
14
21
28
DPI
SFTSV 04
SFTSV 05

(g)
