# Supplementary material for: Pre-existing immunity to heartland virus protects rhesus macaques from challenge with severe fever with thrombocytopenia syndrome virus
Source: Emerg Microbes Infect. 2026 Sep 23;15(1):2731506. doi: 10.1080/22221751.2026.2731506 (PMC13618116; doi:10.1080/22221751.2026.2731506)
Supplement: Supplementary file 2 [file TEMI_A_2731506_SM1403.zip › Supplemental Figure 2.pdf]

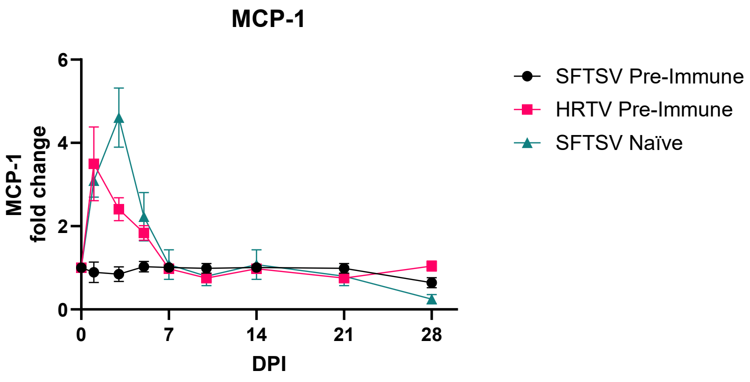
MCP-1
MCP-1
fold change
0
2
4
6
0
7
14
21
28
DPI
SFTSV Pre-Immune
HRTV Pre-Immune
SFTSV Naïve

(a)

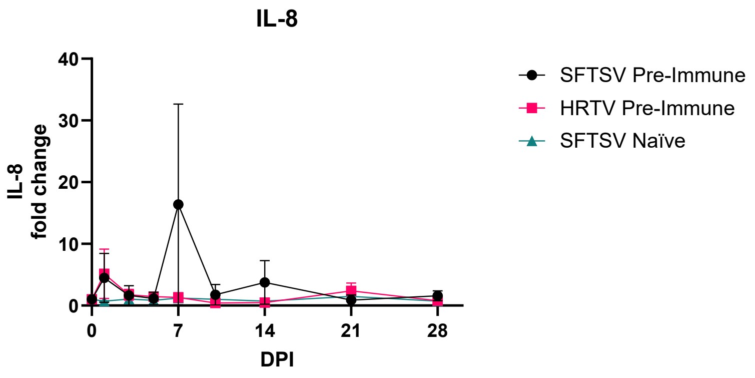
IL-8
IL-8
fold change
0
10
20
30
40
0
7
14
21
28
DPI
SFTSV Pre-Immune
HRTV Pre-Immune
SFTSV Naïve

(b)

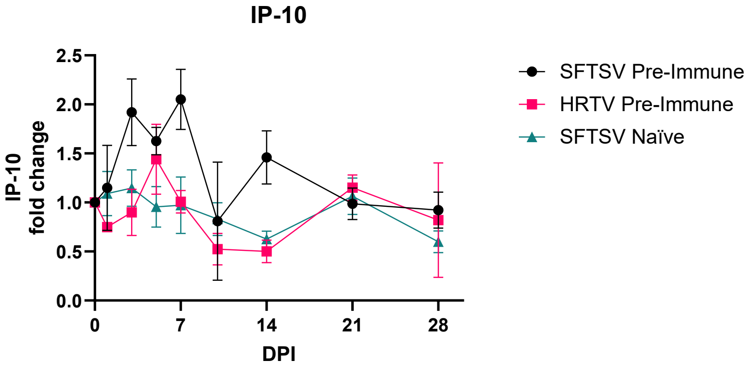
IP-10
IP-10
fold change
0.0
0.5
1.0
1.5
2.0
2.5
0
7
14
21
28
DPI
SFTSV Pre-Immune
HRTV Pre-Immune
SFTSV Naïve

(c)

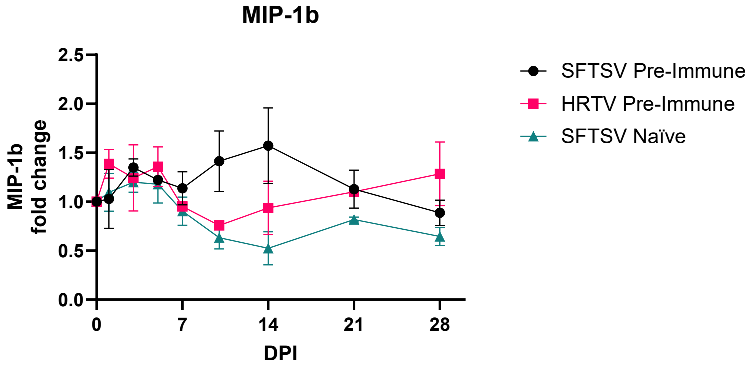
MIP-1b
MIP-1b
fold change
0.0
0.5
1.0
1.5
2.0
2.5
0
7
14
21
28
DPI
SFTSV Pre-Immune
HRTV Pre-Immune
SFTSV Naïve

(d)

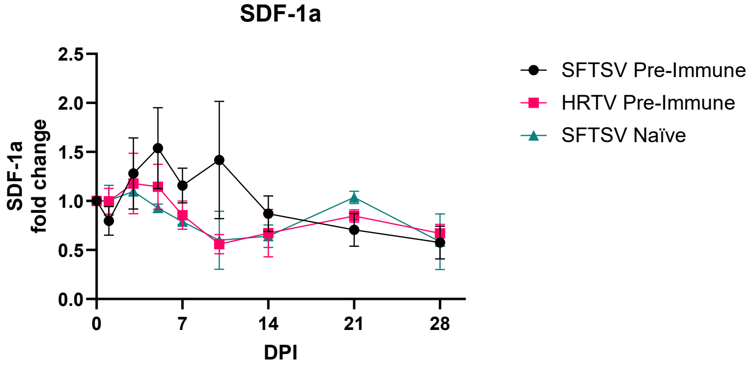
SDF-1a
SDF-1a
fold change
0.0
0.5
1.0
1.5
2.0
2.5
0
7
14
21
28
DPI
SFTSV Pre-Immune
HRTV Pre-Immune
SFTSV Naïve

(e)
