# Supplementary material for: Pre-existing immunity to heartland virus protects rhesus macaques from challenge with severe fever with thrombocytopenia syndrome virus
Source: Emerg Microbes Infect. 2026 Sep 23;15(1):2731506. doi: 10.1080/22221751.2026.2731506 (PMC13618116; doi:10.1080/22221751.2026.2731506)
Supplement: Supplementary file 2 [file TEMI_A_2731506_SM1403.zip › Supplemental Table 1.pdf]

NHP Study ID	**Challenge Virus**	**NHP Sex**	**NHP Age**	**NHP Starting Weight**
**SFTSV 01**	SFTSV SPL010A	Female	~16.5 Years	9.63 kg
**SFTSV 02**	SFTSV SPL010A	Male	~5.9 Years	8.45 kg
**SFTSV 03**	SFTSV SPL010A	Male	~4.2 Years	6.11 kg
**SFTSV 04**	SFTSV YL-1	Female	~17.0 Years	8.72 kg
**SFTSV 05**	SFTSV YL-1	Male	~ 3.8 Years	6.92 kg
**HRTV 01**	HRTV MO-4	Female	~15.9 Years	7.68 kg
**HRTV 02**	HRTV MO-4	Male	~15.2 Years	15.40 kg
**HRTV 03**	HRTV MO-4	Male	~4.1 Years	8.17 kg
