# Supplementary material for: Pre-existing immunity to heartland virus protects rhesus macaques from challenge with severe fever with thrombocytopenia syndrome virus
Source: Emerg Microbes Infect. 2026 Sep 23;15(1):2731506. doi: 10.1080/22221751.2026.2731506 (PMC13618116; doi:10.1080/22221751.2026.2731506)
Supplement: Supplementary file 2 [file TEMI_A_2731506_SM1403.zip › SFTSV_HRTV_NHP_Figures.pdf]

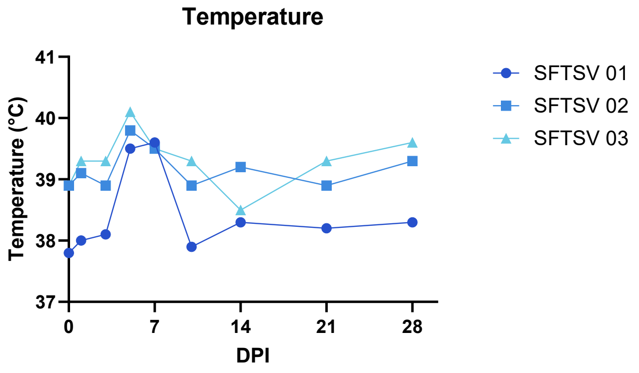
Temperature
Temperature (°C)
41
40
39
38
37
0
7
14
21
28
DPI
SFTSV 01
SFTSV 02
SFTSV 03

(a)

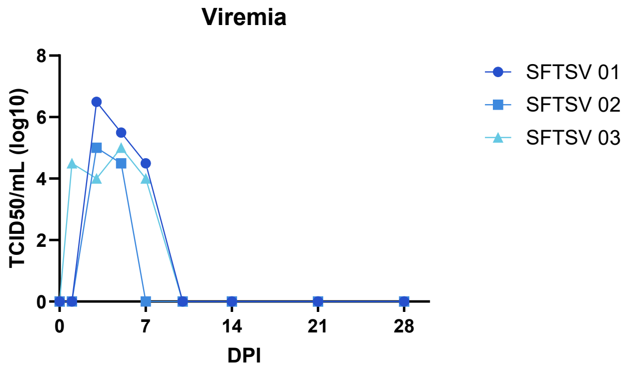
Viremia
TCID50/mL (log10)
8
6
4
2
0
0
7
14
21
28
DPI
SFTSV 01
SFTSV 02
SFTSV 03

(b)

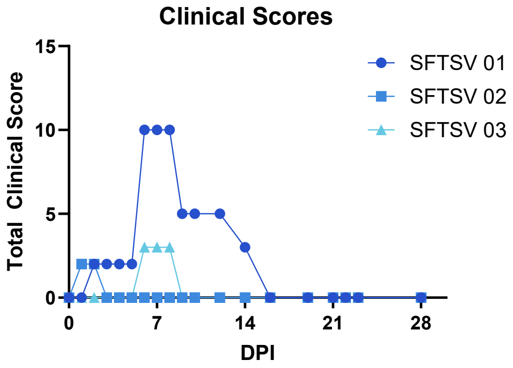
Clinical Scores
Total Clinical Score
15
10
5
0
0
7
14
21
28
DPI
SFTSV 01
SFTSV 02
SFTSV 03

(c)

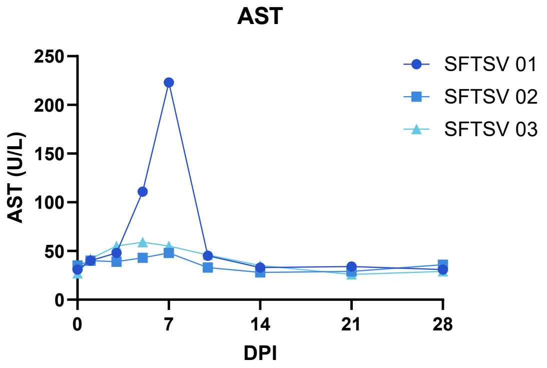
AST
AST (U/L)
250
200
150
100
50
0
0
7
14
21
28
DPI
SFTSV 01
SFTSV 02
SFTSV 03

(d)

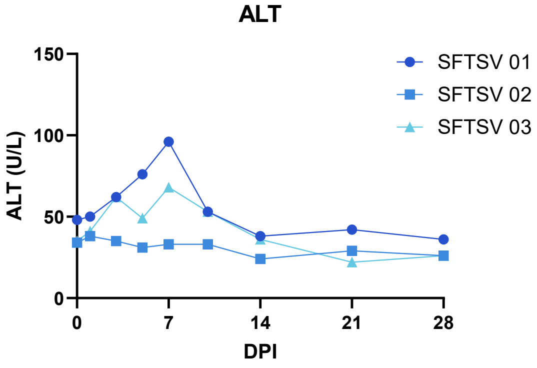
ALT
ALT (U/L)
150
100
50
0
0
7
14
21
28
DPI
SFTSV 01
SFTSV 02
SFTSV 03

(e)

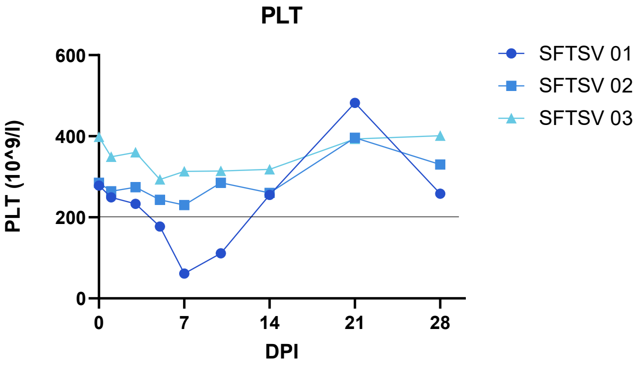
PLT
PLT (10^9/l)
600
400
200
0
0
7
14
21
28
DPI
SFTSV 01
SFTSV 02
SFTSV 03

(f)

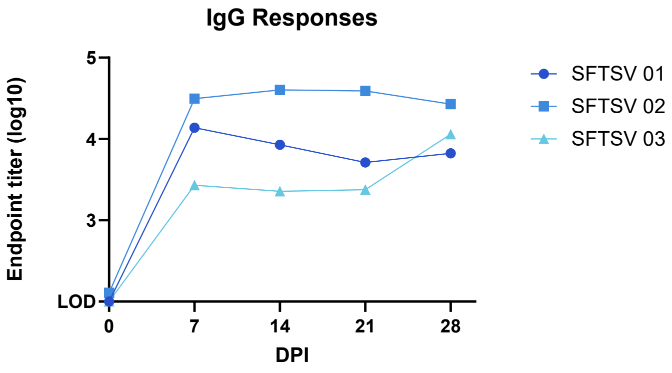
IgG Responses
Endpoint titer (log10)
5
4
3
LOD
0
7
14
21
28
DPI
SFTSV 01
SFTSV 02
SFTSV 03

(g)

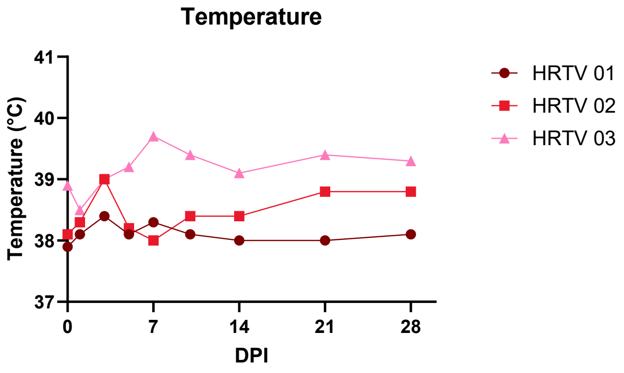
Temperature
Temperature (°C)
41
40
39
38
37
0
7
14
21
28
DPI
HRTV 01
HRTV 02
HRTV 03

(a)

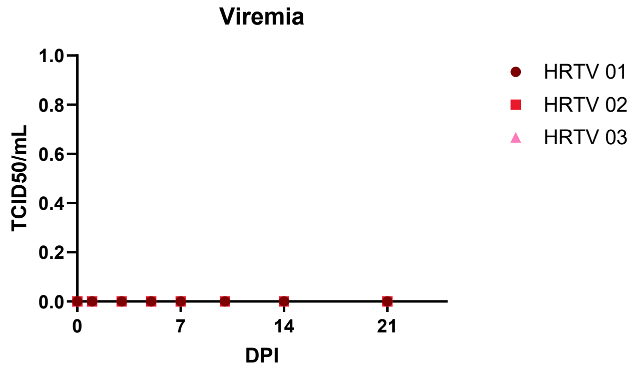
Viremia
TCID50/mL
1.0
0.8
0.6
0.4
0.2
0.0
0
7
14
21
DPI
HRTV 01
HRTV 02
HRTV 03

(b)

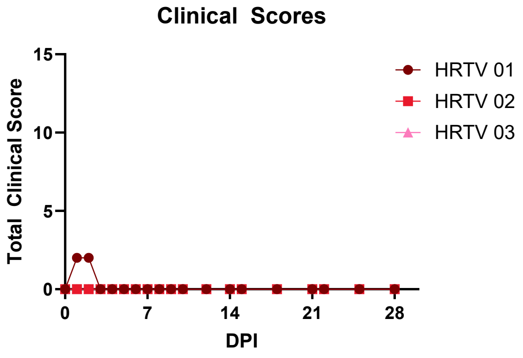
Clinical Scores
Total Clinical Score
15
10
5
0
0
7
14
21
28
DPI
HRTV 01
HRTV 02
HRTV 03

(c)

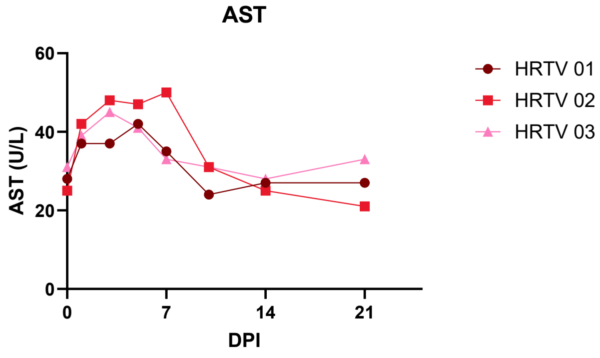
AST
AST (U/L)
60
40
20
0
0
7
14
21
DPI
HRTV 01
HRTV 02
HRTV 03

(d)

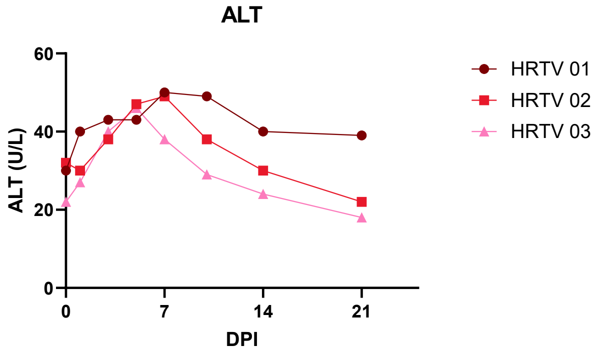
ALT
ALT (U/L)
60
40
20
0
0
7
14
21
DPI
HRTV 01
HRTV 02
HRTV 03

(e)

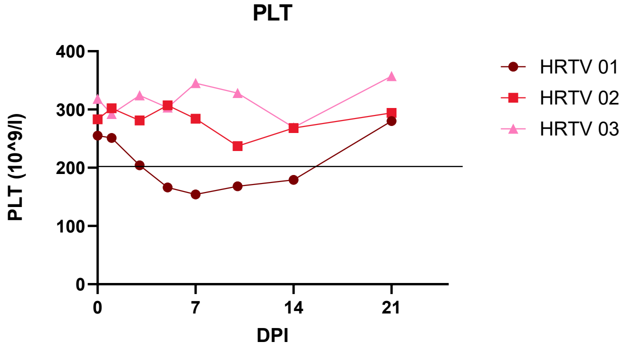
PLT
PLT (10^9/l)
400
300
200
100
0
0
7
14
21
DPI
HRTV 01
HRTV 02
HRTV 03

(f)

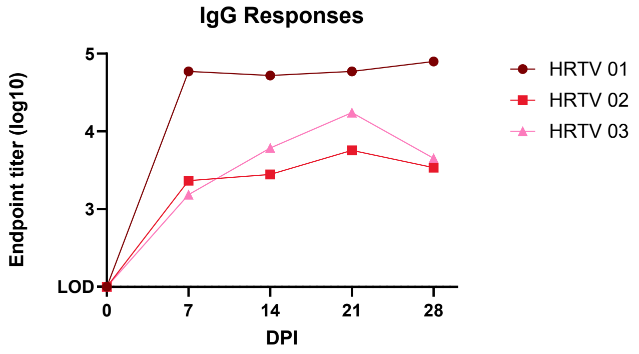
IgG Responses
Endpoint titer (log10)
5
4
3
LOD
0
7
14
21
28
DPI
HRTV 01
HRTV 02
HRTV 03

(g)

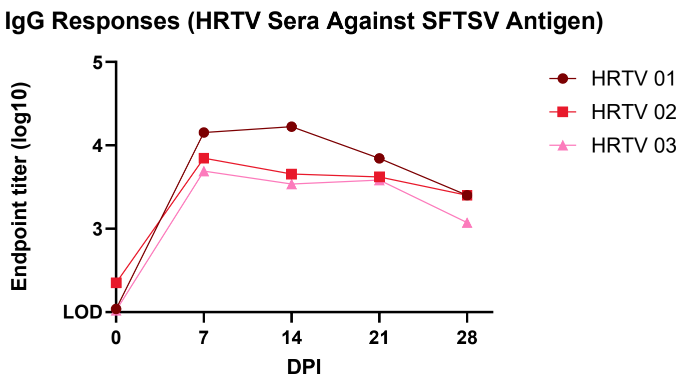
IgG Responses (HRTV Sera Against SFTSV Antigen)
Endpoint titer (log10)
5
4
3
LOD
0
7
14
21
28
DPI
HRTV 01
HRTV 02
HRTV 03

(a)

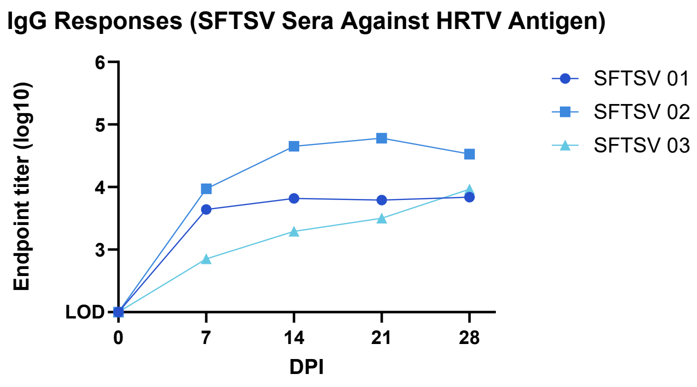
IgG Responses (SFTSV Sera Against HRTV Antigen)
Endpoint titer (log10)
6
5
4
3
LOD
0
7
14
21
28
DPI
SFTSV 01
SFTSV 02
SFTSV 03

(b)

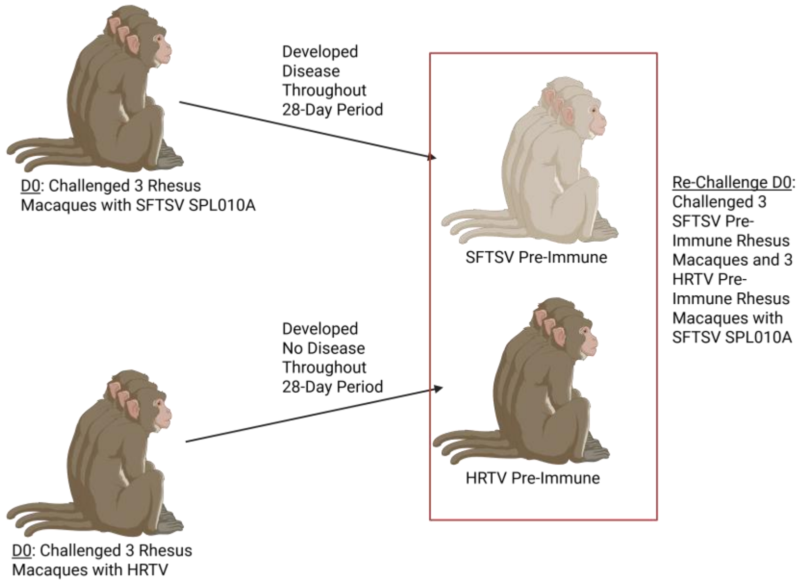
Developed Disease Throughout 28-Day Period
D0: Challenged 3 Rhesus Macaques with SFTSV SPL010A
SFTSV Pre-Immune
Re-Challenge D0: Challenged 3 SFTSV Pre-Immune Rhesus Macaques and 3 HRTV Pre-Immune Rhesus Macaques with SFTSV SPL010A
Developed No Disease Throughout 28-Day Period
HRTV Pre-Immune
D0: Challenged 3 Rhesus Macaques with HRTV

(c)

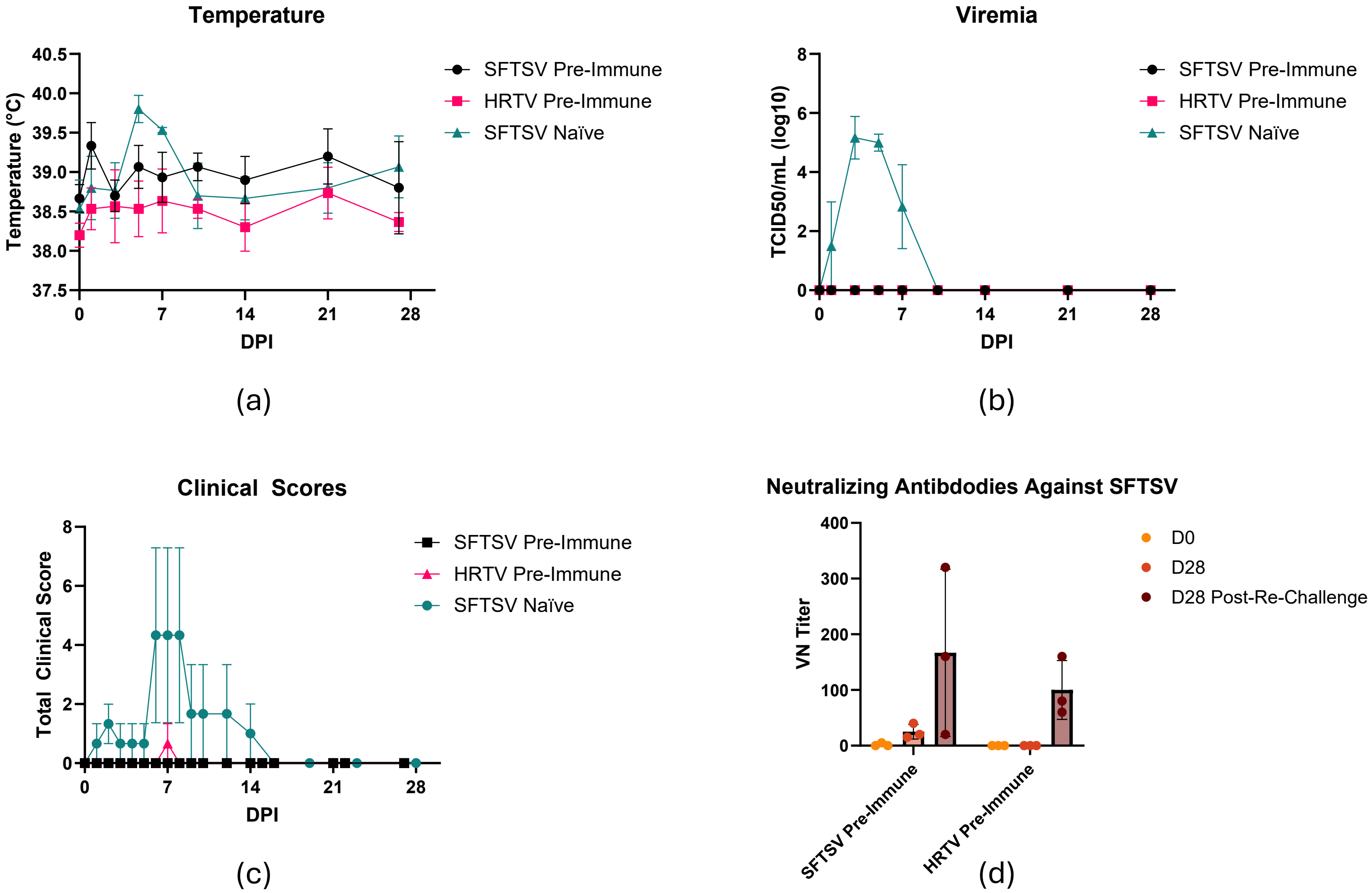

Temperature
Temperature (°C)
40.5
40.0
39.5
39.0
38.5
38.0
37.5
0
7
14
21
28
DPI
SFTSV Pre-Immune
HRTV Pre-Immune
SFTSV Naïve
(a)
Viremia
TCID50/mL (log10)
8
6
4
2
0
0
7
14
21
28
DPI
SFTSV Pre-Immune
HRTV Pre-Immune
SFTSV Naïve
(b)
Clinical Scores
Total Clinical Score
8
6
4
2
0
0
7
14
21
28
DPI
SFTSV Pre-Immune
HRTV Pre-Immune
SFTSV Naïve
(c)
Neutralizing Antibdodies Against SFTSV
VN Titer
400
300
200
100
0
SFTSV Pre-Immune
HRTV Pre-Immune
D0
D28
D28 Post-Re-Challenge
(d)

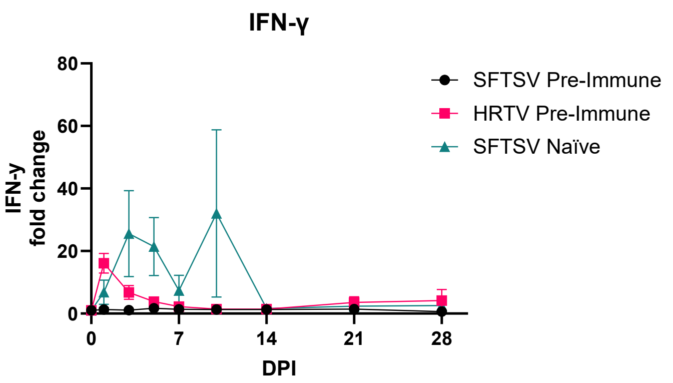
IFN-γ
IFN-γ fold change
80
60
40
20
0
0
7
14
21
28
DPI
SFTSV Pre-Immune
HRTV Pre-Immune
SFTSV Naïve

(a)

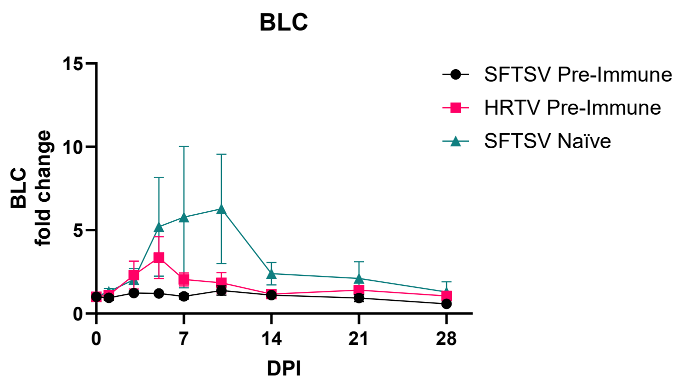
BLC
BLC fold change
15
10
5
0
0
7
14
21
28
DPI
SFTSV Pre-Immune
HRTV Pre-Immune
SFTSV Naïve

(b)

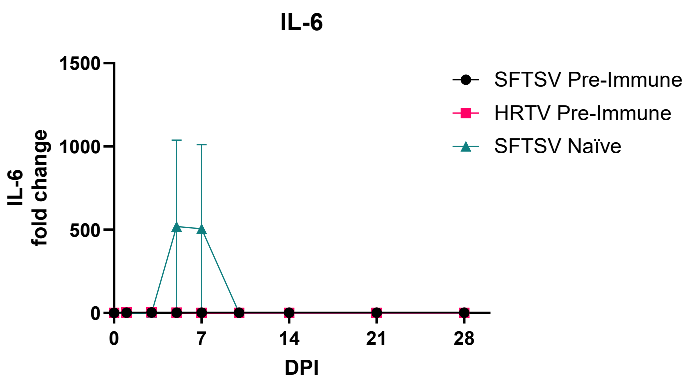
IL-6
IL-6 fold change
1500
1000
500
0
0
7
14
21
28
DPI
SFTSV Pre-Immune
HRTV Pre-Immune
SFTSV Naïve

(c)

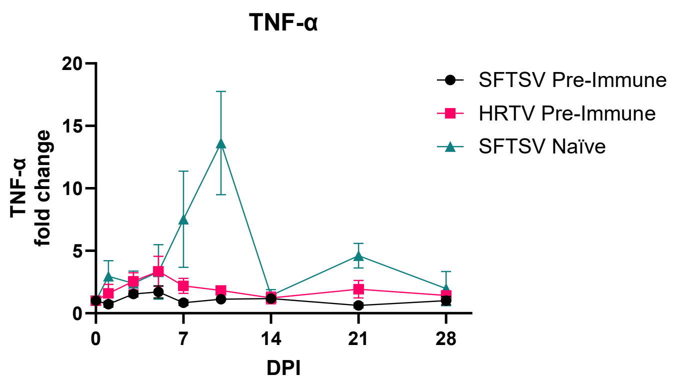
TNF-α
TNF-α fold change
20
15
10
5
0
0
7
14
21
28
DPI
SFTSV Pre-Immune
HRTV Pre-Immune
SFTSV Naïve

(d)

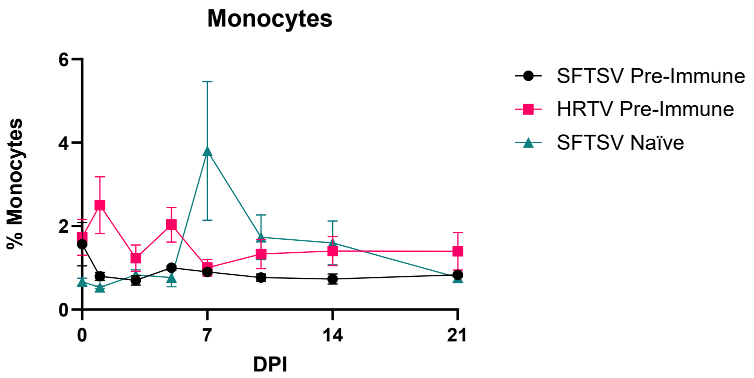
Monocytes
% Monocytes
6
4
2
0
0
7
14
21
DPI
SFTSV Pre-Immune
HRTV Pre-Immune
SFTSV Naïve

(e)
